# A Simple and Versatile Method for Ex Vivo Monitoring of Goat Vaginal Mucosa Transduction by Viral Vector Vaccines

**DOI:** 10.3390/vaccines12080851

**Published:** 2024-07-29

**Authors:** Sergio Minesso, Amienwanlen Eugene Odigie, Valentina Franceschi, Camilla Cotti, Sandro Cavirani, Maria Tempesta, Gaetano Donofrio

**Affiliations:** 1 Department of Veterinary Science, University of Parma, 43126 Parma, Italy; sergio.minesso@unipr.it (S.M.); amienwanlen.odigie@uniba.it (A.E.O.); valentina.franceschi@unipr.it (V.F.); camilla.cotti@studenti.unipr.it (C.C.); sandro.cavirani@unipr.it (S.C.); 2 Department of Veterinary Medicine, University of Bari, 70010 Valenzano, Italy; maria.tempesta@uniba.it

**Keywords:** viral vectors, bioluminescent imaging, vaginal mucosa vaccines, genital diseases, goat, animal model

## Abstract

Goat may represent a valid large animal model for human pathogens and new vaccines testing. Appropriate vaccine administration is a critical component of a successful immunization program. The wrong route of administration may reduce the efficacy of the vaccine, whereas the proper administration strategy can enhance it. Viral vectors have been employed successfully for goat and sheep immunization; however, no data concerning the vaginal route are available. A viral vector’s ability to transduce the site of inoculation is of primary interest. In this study, a fast and reliable ex vivo assay for testing the transduction capability of an Ad5-based vector when intravaginally administered was developed. An Ad5 vector delivering an expression cassette with a bicistronic reporter gene, Ad5-CMV-turboGFP-IRES-Luc2, was constructed. We demonstrated Ad5-CMV-turboGFP-IRES-Luc2’s ability to transduce caprine vaginal mucosa by ex vivo bioluminescent imaging (BLI) employing a simple CCD camera apparatus for chemiluminescence western immunoblotting. These data, though simple, provide valuable insights into developing a vaginal immunization strategy using a viral vector-based vaccine to protect against pathogens causing genital diseases.

## 1. Introduction

According to WHO estimates, roughly one million sexually transmitted infections are contracted daily by women, mainly from marginalized areas of poor countries (https://www.who.int/news-room/fact-sheets/detail/sexually-transmitted-infections-(stis), accessed on 30 May 2024). These infections are associated not only with reproductive complications, but also with death. Although effective therapeutic interventions are available, the logistical and economic inaccessibility of treatment, as well as a lack of infrastructures and social stigmatization, increase disease severity. Thus, prevention could represent an effective method to overcome limitations associated with therapy. Vaccination represents the most effective transmissible diseases prevention method. For instance, the human papillomavirus (HPV) vaccine campaign saved thousands of lives from cervical cancer [1]. The vagina is the main pathway of entry for sexually transmitted viruses and bacteria, and vaginal mucosa immunity serves as the first line of defense. Thus, intravaginal vaccination could represent a favorable strategy to promote a protective immunity of the genital tract. Exposure of the vaginal mucosa to specific pathogen antigens could generate first an innate, and later an adaptive, immune response, favoring an immune memory in the draining lymph nodes, and thereby leading to a faster and effective pathogen clearance [1,2]. A key prerequisite for a successful vaccination is the antigen delivery system employed, and viral vectors are the most promising option [3,4,5,6,7,8]. Several reports have documented the induction of both local and systemic immune responses after vaginal administration of different viral vector-based vaccines against STIs [1]. For instance, a vaginal prime/boost strategy employing lipopeptide/adenovirus type 5, delivering a herpes simplex virus-2 (HSV-2) immunodominant peptide, resulted in a durable mucosal CD8+ response that protected mice in an HSV-2 lethal challenge [1]. Using viruses as platforms for delivering heterologous antigens requires careful consideration due to the immune system’s advanced mechanisms for detecting and eliminating viruses. A viral vector introduces the antigen directly into the host cell, allowing robust intracellular expression and acting as both a delivery system and an adjuvant. For a viral vector to be effective, it must present the heterologous antigen as an immune target and persist in the host long enough to elicit an effective response. Therefore, transduction efficiency at the inoculation site of a viral vector is a crucial issue. In the present study, a simple and versatile method to monitor vaginal mucosa transduction by a viral vector was developed in a large animal model, the goat, for genital herpes.

## 2. Materials and Methods

### 2.1. Cells

HEKs (human embryo kidney cells) 293 T (ATCC: CRL-11268) were grown in complete Eagle’s minimal essential medium (cEMEM: 1 mM of sodium pyruvate, 2 mM of L-glutamine, 100 IU/mL of penicillin, 100 μg/mL of streptomycin, and 0.25 μg/mL of amphotericin B), supplemented with 10% FBS, and incubated at 37 °C/5% CO_2_ in a humidified incubator. Caprine fetal kidney primary cells (CFKs) were obtained from caprine fetal kidney as previously described [9], with modifications. Briefly, a caprine fetus was collected at a commercial abattoir and transported to the laboratory on ice. The kidney was first isolated aseptically and washed twice with phosphate buffered saline (PBS). Next, the capsule was removed and 2–3 mm^3^ pieces of cortical tissues were collected, washed twice in PBS, and further minced using a sterile scalpel. The minced tissue was then trypsinized for 30 min at 37 °C under constant stirring. The supernatant was harvested and centrifuged at 1000 rpm (~170× *g*) for 10 min. The resulting cell pellet was resuspended in cEMEM with 10% FBS. Finally, diluted cell suspension was dispensed in 25 cm^2^ flasks at a density of 0.3 × 10^6^ cells/flask and maintained at 37 °C with 5% CO_2_. All supplements for the culture medium were purchased from Gibco (Gibco, Segrate, MI, Italy).

### 2.2. Adenovirus Reconstitution

pAd5-CMV-TurboGFP-IRES-Luc2 was chemically synthesized (VectorBuilder), assessed by sequencing, and then reconstituted as previously described [10,11,12,13]. Briefly, HEK 293 T cells were seeded into 25 cm^2^ flasks (1 × 10^6^ cells/flask). When cells’ monolayers reached sub-confluence, the medium was removed, and a Polyethylenimine (PEI) transfection reagent (Polysciences, Inc., Warrington, PA, USA) was used to transfect cells with pAd5-CMV-TurboGFP-IRES-Luc2. Briefly, 3 µg of pAd5-CMV-TurboGFP-IRES-Luc2 was linearized using PacI (Thermoscientific, Waltham, MA, USA) restriction enzyme and mixed with 7.5 µg of PEI (1 mg/mL) (ratio 1:2.5 D PEI) in 500 µL of Dulbecco’s modified essential medium (DMEM) high glucose (Euroclone, Pero, MI, Italy) without serum. The transfection mixture was incubated for 15 min at room temperature and 2000 µL of DMEM was added before transferring the transfection solution onto cells’ monolayers. Cells were then incubated for 6 h at 37 °C with 5% CO_2_. Next, the transfection mixture was replaced with fresh cEMEM containing 10% FBS. When CPE affected ~50% of the cell monolayer, the flasks were frozen at −80 °C, thawed, and the culture supernatant was harvested. The supernatant containing viral particles was then clarified by centrifugation at 3500 rpm (~2000× *g*) for 10 min at 4 °C and filtered using a 0.45 µm filter.

### 2.3. Adenoviral Vector Growth and Tittering

HEK293T cells were seeded in 25 cm^2^ flasks (0.5 × 10^6^ cells/flask) and incubated at 37 °C with 5% CO_2_. When cells reached sub-confluence, the culture medium was removed, and cells were infected with 0.5 mL of the supernatant harvested during the viral particles’ reconstitution. Flasks were frozen when the CPE affected ~50% of the cell monolayer, and after thawing, the clarified and filtered supernatant was tittered. Viral titration was performed by infecting HEK293T with a 10-fold dilution of Ad5-CMV-TurboGFP-IRES-Luc2. The number of transducing units (TUs)/mL was estimated 3 days post infection. Ad5-CMV-TurboGFP-IRES-Luc2 was then amplified, infecting HEK 293T cells at a multiplicity of infection (MOI) of 0.1. Cells were checked daily for CPE and fluorescence evaluation, and 0.5 mL of cell supernatant was harvested at 24 and 48 h post infection for further tittering.

### 2.4. Caprine Foetal Kidney Primary Cells Transduction

CFK cells at the 5th passage were seeded into a 6-well plate at a density of 0.3 × 10^6^ cells/well and incubated at 37 °C with 5% CO_2_. When cells were sub-confluent, the culture medium was removed, and cells were infected with Ad5-CMV-turboGFP-IRES-Luc2 at MOIs of 1 and 10. Twenty-four hours pi cells were observed using inverted fluorescence microscopy (Zeiss-Ax, iovert-S100, Zeiss, Oberkochen, Germany) to monitor GFP expression. The transduction efficiency of CFK was measured as the percentage of cells expressing GFP assessed by flow cytometry analysis with a FACS Canto II (BD Biosciences, Milan, MI, Italy). Briefly, CFK cells were detached with trypsin, extensively washed two times in PBS, and resuspended in cold PBS prior to cytometric analyses. Fluorescence intensity was determined with 40,000 cells per sample using a gating strategy for GFP signals based on the background signal of the untransduced CFK. Data acquisition and analysis were performed with Diva 9.02 software (BD Bioscience, Milan, MI, Italy).

### 2.5. Goat Vagina Organotypic Culture Establishment and Transduction

The complete reproductive tracts of two adult goats were collected under sterile conditions at a commercial abattoir, transported to the lab on ice, and immediately processed. Vaginas were isolated from surrounding tissues and ~2 cm^2^ full thickness pieces were obtained from the middle third of the organ (~2 cm from the cervix and the external vaginal orifice) using a sterile scalpel. These pieces were then quickly dipped in sterile PBS containing 50 mg/mL of gentamicin (Gibco) and placed in a 6-well plate with the mucosal surface facing upwards. Wells were then filled with 3 mL of cEMEM with 10% FBS containing 10^6^ transducing units (TUs) of Ad5-CMV-turboGFP-IRES-Luc2, or cEMEM with 10% FBS as a negative control. Organotypic cultures were incubated at 37 °C/5% CO_2_.

### 2.6. Bioluminescent Imaging (BLI) and Quantification

CFK cells at the 5th passage were seeded into a 6-well plate at a density of 0.3 × 10^6^ cells/well and incubated at 37 °C with 5% CO_2_. When cells were sub-confluent, the culture medium was removed, and cells were infected with Ad5-CMV-turboGFP-IRES-Luc2 at MOIs of 1 and 10. Twenty-four hours pi the cell supernatant was replaced with fresh cEMEM containing D-luciferin (Perkin Elmer, Milan, MI, Italy, 1 mg/mL in 0.9% sodium chloride), and bioluminescent imaging of firefly LUC was performed using the CCD camera apparatus (ChemiDoc XRS+, BioRad, Segrate, MI, Italy). The plate was then exposed using standard chemiluminescence detection settings (signal accumulation mode) and visualized in grey and pseudocolor scale. For ex vivo analysis, cEMEM containing D-luciferin was gently poured onto the vagina mucosal surface 72 h pi. Plates were then exposed to CCD camera apparatus, as for cell monolayers. Image quantification was implemented using Python version 3.9.18, (https://docs.python.org/3/reference/index.html, accessed on 25 April 2024). Achieving this task involved the initiation of several core libraries and their respective dependencies in the Python programming environment. These imported libraries provided necessary functionalities to achieve image manipulation, analysis, and visualization. They included Open-Source Computer Vision Library (OpenCV) with capabilities for image processing, Pandas for high-performance data analysis, Numerical Python (NumPy) for array operations [14], and Matplotlib and Seaborn for interactive and statistical data visualizations. The imported positive bioluminescent and negative control images were grayscale-transformed to two-dimensional NumPy arrays for ease of computation, and a threshold cutoff value of 32 was applied using the threshold-binary-inverse method. Hence, pixels with intensity values equal to or greater than the threshold were set as zero (0), while intensity values less than the threshold were set as a maximum value of 255. Concurrently, the roles of black and white were reversed. Next, the chain-approximate-simple method was applied to compress horizontal, vertical, and diagonal segments of each identified external contour boundary of all retrieved thresholder-transformed images. Contour approximations were outputted as a NumPy array of x- and y-coordinates of contour points [14]. Finally, the combined area of all estimated contours for each image, an approximation of the measure of the intensity of bioluminescence, was calculated in pixel squared (psq) units. The complete code implementation and visualizations are provided in Supplementary File S1.

## 3. Results and Discussion

**Figure 1:**
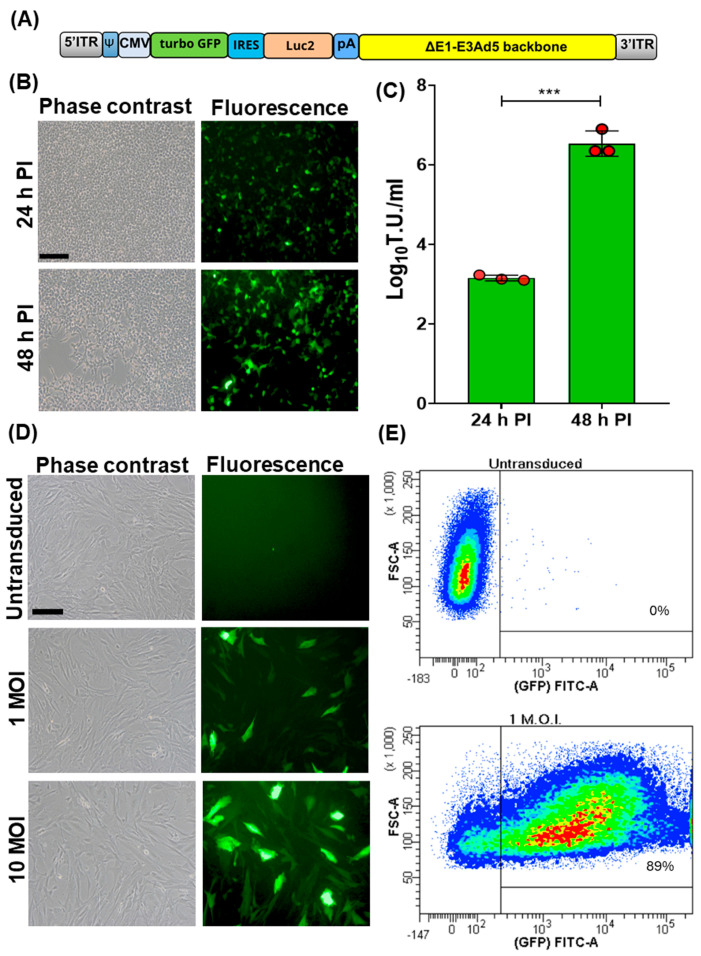
Generation and transduction efficiency of Ad5-CMV-turboGFP-IRES-Luc2. (**A**) Schematic diagram (not to scale) of Ad5-CMV-turboGFP-IRES-Luc2 genomic composition: 5′ and 3′ inverted terminal repeat (ITR; grey), adenoviral packaging signal (Ψ; blue), human cytomegalovirus immediate early enhancer/promoter (CMV; azure), open reading frame coding for turbo GFP (green), internal ribosomal entry site (IRES; turquoise), human codon usage adapted luciferase ORF (Luc2; orange), bovine growth hormone polyadenylation signal (pA; blue), and E1A/B and E3 deleted human adenovirus type 5 genome backbone (ΔE1-E3Ad5 backbone; yellow). (**B**) Phase contrast and fluorescence images of Ad5-CMV-turboGFP-IRES-Luc2 infected HEK293T (scale bar corresponds to 100 µm) at 24 and 48 h post infection (pi). (**C**) Respective viral titters expressed as log10 per mL of transducing units (TUs) of viral particles released at 24 and 48 h pi (*** *p* < 0.01). Values are the means ± standard errors of three independent experiments. (**D**) Phase contrast and fluorescence images of Ad5-CMV-turboGFP-IRES-Luc2 transduced fetal caprine kidney primary cell cultures at different MOIs (1 and 10) and the untransduced control. (**E**) Representative images of the efficiency of transduction, as quantified by cytofluorimetric analysis, when cells were transduced with 1 MOI of Ad5-CMV-turboGFP-IRES-Luc2; 89% transduction efficiency was obtained with respect to the untransduced control. The experiment was repeated three times with similar results.

**Figure 2:**
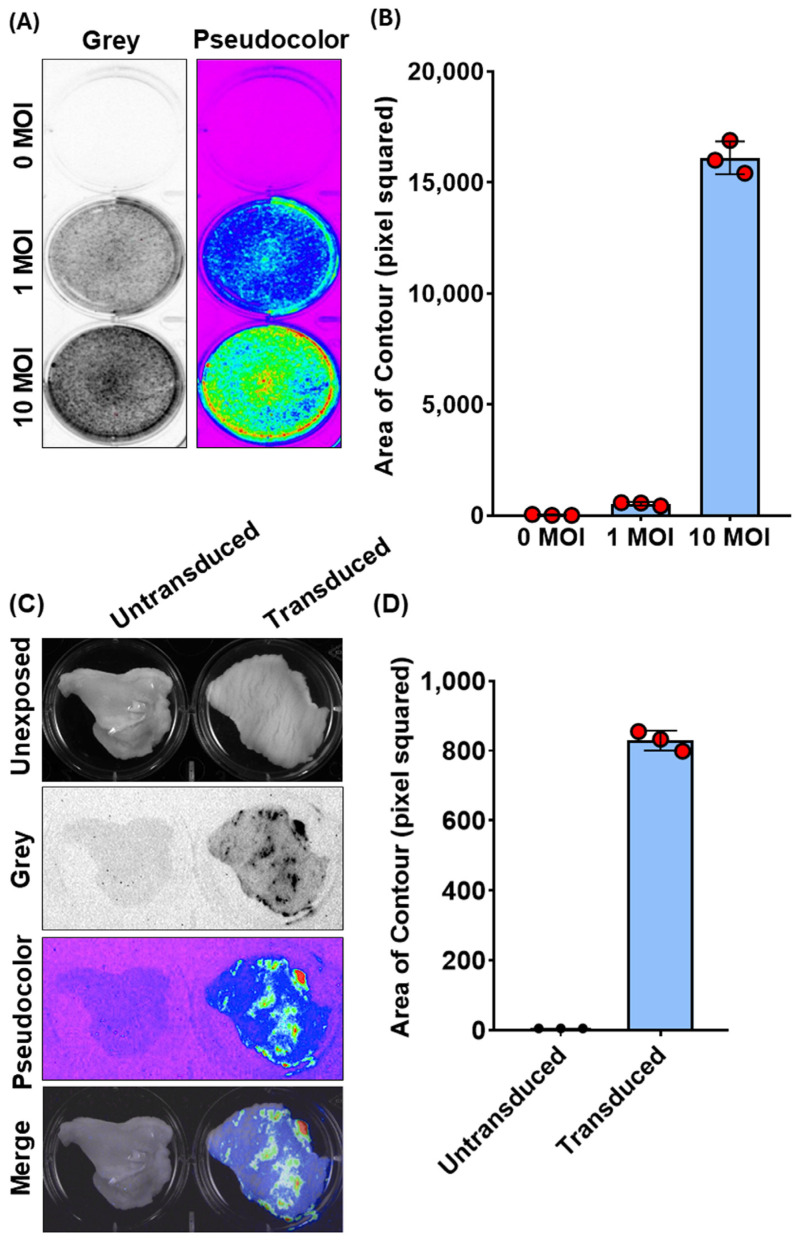
Bioluminescence imaging (BLI) and quantification of Ad5-CMV-turboGFP-IRES-Luc2 transduction in CFK cells and vaginal organotypic cultures. (**A**) Representative images of Ad5-CMV-turboGFP-IRES-Luc2 transduced (1 and 10 MOIs) and untransduced (0 MOI) CFK cells’ monolayers expressing luciferase, as observed by BLI in grey or pseudocolor mode and (**B**) their respective quantification expressed as pixel squared (Supplementary File S1). Data presented are the means ± standard errors of triplicate measurements (measured by the Student’s *t*-test). (**C**) Representative BLI images of Ad5-CMV-turboGFP-IRES-Luc2 transduced and untransduced vaginal organotypic cultures, as observed by BLI in grey or pseudocolor mode, as well as their unexposed and merge images. (**D**) Photon emissions, quantified as in (**B**). Data presented are means ± standard errors of triplicate measurements (measured by the Student’s *t*-test).

A bicistronic replicating incompetent human adenovirus type 5, Ad5-TurboGFP-IRES-Luc2, was generated [10,11,12,13]. TurboGFP ORF, also known as maxGFP (green fluorescent protein from maxillopoda) [15], considered the highest in brightness, photo- and pH-stability among all fluorescent proteins, was linked to the 5′ end of the encephalomyocarditis virus internal ribosome entry site (IRES) [16]. This allowed the initiation of translation internally on a transcript regardless of its 5′ end. TurboGFP-IRES was provided at the 3′ end of a humanized firefly luciferase ORF (Luc2) [17], which was codon usage optimized to reduce cryptic transcription factor binding sites. The bicistronic TurboGFP-IRES-Luc2 ORF was then placed under the transcriptional control of the human cytomegalovirus (CMV) immediate early promoter and the herpes simplex virus thymidine kinase polyadenylation signal and integrated into a replicating incompetent type 5 adeno viral transfer vector (Ad5) backbone (Figure 1A). Ad5-TurboGFP-IRES-Luc2 infectious particles were reconstituted by transient transfection in HEK cells. These constitutively express E1A and E1B genes, thus conferring replication competence to Ad5-TurboGFP-IRES-Luc2, which is lost in cells lacking E1A and E1B expression. In fact, CPE and an increase in viral titer was observed only in HEK293 cells (Figure 1B,C). Initially Ad5-TurboGFP-IRES-Luc2’s transduction ability was evaluated on fetal caprine kidney primary cell cultures. Cells infected with Ad5-TurboGFP-IRES-Luc2 expressed both GFPs, as monitored by fluorescence microscopy and cytofluorimetric analysis (Figure 1D,E), and luciferase, as monitored by chemiluminescence imaging using a CCD camera apparatus for chemiluminescence detection of protein expression by western immunoblotting (Figure 2A,B).

Regarding this latter observation, we wanted to determine if the same method could be used to detect chemiluminescence in the whole tissue, specifically the goat vaginal mucosa. Although Ad vectors have been successfully employed via subcutaneous or intramuscular vaccination in the goat, no data are available regarding its capability to transduce the vaginal mucosa [18,19]. Therefore, we attempted to use chemiluminescence imaging to monitor Ad5-TurboGFP-IRES-Luc2 in an ex vivo setting. Vaginas were collected in sterile conditions from slaughtered goats at the abattoir, transported to the lab on ice, and immediately processed. Approximately 2 cm^2^ pieces of tissue were obtained from the middle third of the organ and placed in a 6-well plate with the mucosal surface facing upwards. Wells were filled with complete medium containing 10^6^ transducing units (TUs) of Ad5-CMV-turboGFP-IRES-Luc2 and incubated at 37 °C/5% CO_2_. Seventy-two hours post infection, luciferin substrate was added to each well, and the plate was exposed to the CCD camera apparatus (ChemiDoc XRS+, BioRad). Indeed, it was possible to visualize ex vivo Ad5-CMV-turboGFP-IRES-Luc2 gene delivery on the vaginas’ mucosa surface as shown by the chemiluminescent emission from treated samples compared to the untreated control (Figure 2C). Moreover, it was possible to quantify the signal intensity (Figure 2D). The complete code implementation and visualizations are provided in File S1. Our quantification method was consistent with the subjective visual evaluation of bioluminescent intensity using CCD camera apparatus (ChemiDoc XRS+, BioRad). These data, though simple, provide valuable insights into developing a vaginal immunization strategy using a viral vector-based vaccine to protect against pathogens causing genital diseases.

Mammalians’ vaginas are fibromuscular ducts extending from the vulva to the cervix, connecting the external environment to the uterus. Vaginal mucosa is lined by a non-keratinized squamous epithelium, apically covered by cornified cells permeable to bacteria and viruses and embedded by cells and molecules belonging to the innate and acquired immune systems, thereby playing a dual role in acquiring and combatting pathogens. The vagina is drained by the iliac and inguinal lymph nodes. These biological milieus confer to the vagina the right characteristics to be an inductive site of cell and humoral immunity and an interesting site for vaccine administration to induce effective protection against sexually transmitted pathogens [1,20]. A suitable animal model is necessary to incorporate early research findings into preclinical development. Mice and guinea pigs are normally used as animal models for human diseases [21,22]; however, none of these animal infection systems is a perfect model for sexually transmitted diseases. Indeed, small animal models may have inconstant success in predicting vaccine efficacy in larger animals, whereas larger animal models have been shown to predict vaccine outcomes more accurately in humans [23]. From this perspective, caprine herpes virus-1 (CpHV-1) infection in goats shares several biological similarities with herpes simplex virus-2 (HSV-2) infection in humans. Both viruses have preferential tropism for the genital area, causing vesicular/ulcerative lesions and establishing latency in the sacral ganglia [24,25,26,27]. CpHV-1 infection in goats may offer an excellent model exploitable for pathogenic studies, antiviral molecules, and new vaccines testing. Also, the infection is methodically reproducible; clinical scoring of the infection is simple to evaluate; and lesions can be easily graded [27]. Moreover, a goat is an easy animal to approach, its housing is low-cost, and blood drawings as well as tissue collection are easy to perform. Different recombinant vectors functionalized with CpHV-1 immunodominant glycoproteins can be administered vaginally or with the “prime and pull” strategy to evaluate both immunity and protection. The demonstration of the immunogenicity of a recombinant vaccine or the effectiveness of antiviral molecules in the goat model is an important milestone to create immunological correlates to predict protection; thus, it may reduce the time necessary in the rational progression of clinical testing of HSV-2 candidate vaccines or therapeutics in humans. Although replication-defective human serotype 5 adenovirus has been employed successfully as vaccines in goats [18,19], no data are available regarding their intravaginal use. Determining the host/tissue tropism of a viral vector usually requires time- and resources-consuming experiments involving primary cell isolation, in vivo studies, and nucleic acid or protein extraction [28]. Our goal was to develop a rapid assay to effectively monitor the potential of a vaccine administration route before attempting in vivo experimentation. Our assay enabled the direct monitoring of viral transduction after ex vivo infection, utilizing a simple CCD camera apparatus in a time- and cost-efficient manner.

## 4. Conclusions

In the present study, we developed a reliable assay for ex vivo monitoring of goat vaginal mucosa transduction. Ad5-CMV-turboGFP-IRES-Luc2 successfully transduced goat vaginal mucosa, supporting the potential intravaginal administration of an Ad5 based vaccine. The present data can be considered a simplified starting platform for further investigation, not only of Ad5-based vectors, but also of other viral vectors vaccines or therapeutics for sexually transmitted diseases, extendible to other animals and humans.

## Data Availability

All data are available in this paper and Supplementary Materials, which can be retrieved by clicking the dedicated link.

## Supplementary Materials

The following supporting information can be downloaded at: https://www.mdpi.com/article/10.3390/vaccines12080851/s1, File S1: Code implementation and visualization.
