# Supplementary material for: A Simple and Versatile Method for Ex Vivo Monitoring of Goat Vaginal Mucosa Transduction by Viral Vector Vaccines
Source: Vaccines (Basel). 2024 Jul 29;12(8):851. doi: 10.3390/vaccines12080851 (PMC11359855; doi:10.3390/vaccines12080851)

CODE IMPLEMENTATION AND VISUALIZATION

In [1]:

```
"""
The following libraries and its respective dependencies are imported into the
Python programming environment including:
Numerical Python (NumPy) as np for array operations
Matplotlib and Seaborn for data visualization
Pandas for data analysis
Open-Source Computer Vision (cv2) for image processing
"""

# import all libraries
import numpy as np
import matplotlib.pyplot as plt
import seaborn as sns
import pandas as pd
import cv2
```

STEPS IN ACTUAL QUANTIFICATION

Create Image Path

In [2]:

```
"""
File path containing the respective images for analysis are assigned different
variables names. The first image set, second
image set and luciferase intensity represents three subsets of image categories
used in the analysis and result validation
"""

# identify file path
# First image set
img_path1a = r"C:\Users\Utente\OneDrive\Desktop\My
folder\Projects\Professor_DINOFRIO_UNIPR\Mini_projects\vaginabioluiminescentimag
ing\single_img\neg_r1a.tif"
img_path1b = r"C:\Users\Utente\OneDrive\Desktop\My
folder\Projects\Professor_DINOFRIO_UNIPR\Mini_projects\vaginabioluiminescentimag
ing\single_img\neg_r1b.tif"

img_path1c = r"C:\Users\Utente\OneDrive\Desktop\My
folder\Projects\Professor_DINOFRIO_UNIPR\Mini_projects\vaginabioluiminescentimag
ing\single_img\pos_r1c.tif"
img_path2a = r"C:\Users\Utente\OneDrive\Desktop\My
folder\Projects\Professor_DINOFRIO_UNIPR\Mini_projects\vaginabioluiminescentimag
ing\single_img\pos_r2a.tif"

img_path2b = r"C:\Users\Utente\OneDrive\Desktop\My
folder\Projects\Professor_DINOFRIO_UNIPR\Mini_projects\vaginabioluiminescentimag
ing\single_img\neg_r2b.tif"
```

```
img_path2c = r"C:\Users\Utente\OneDrive\Desktop\My
folder\Projects\Professor_DINOFRIO_UNIPR\Mini_projects\vaginabioluiminescentimag
ing\single_img\neg_r2c.tif"

# Second image set
img_pathR1 = r"C:\Users\Utente\OneDrive\Desktop\My
folder\Projects\Professor_DINOFRIO_UNIPR\Mini_projects\vaginabioluiminescentimag
ing\single_img\pos_R1.tif"
img_pathR2 = r"C:\Users\Utente\OneDrive\Desktop\My
folder\Projects\Professor_DINOFRIO_UNIPR\Mini_projects\vaginabioluiminescentimag
ing\single_img\neg_R2.tif"

# Luciferase intensity
img_path1A = r"C:\Users\Utente\OneDrive\Desktop\My
folder\Projects\Professor_DINOFRIO_UNIPR\Mini_projects\vaginabioluiminescentimag
ing\single_img\FC_r1a.tif"
img_path1B = r"C:\Users\Utente\OneDrive\Desktop\My
folder\Projects\Professor_DINOFRIO_UNIPR\Mini_projects\vaginabioluiminescentimag
ing\single_img\FP_r1b.tif"
img_path1C = r"C:\Users\Utente\OneDrive\Desktop\My
folder\Projects\Professor_DINOFRIO_UNIPR\Mini_projects\vaginabioluiminescentimag
ing\single_img\FP_r1c.tif"
img_path2A = r"C:\Users\Utente\OneDrive\Desktop\My
folder\Projects\Professor_DINOFRIO_UNIPR\Mini_projects\vaginabioluiminescentimag
ing\single_img\FC_r2a.tif"
img_path2B = r"C:\Users\Utente\OneDrive\Desktop\My
folder\Projects\Professor_DINOFRIO_UNIPR\Mini_projects\vaginabioluiminescentimag
ing\single_img\FP_r2b.tif"
img_path2C = r"C:\Users\Utente\OneDrive\Desktop\My
folder\Projects\Professor_DINOFRIO_UNIPR\Mini_projects\vaginabioluiminescentimag
ing\single_img\FP_r2c.tif"
```

Read Image as BGR and Equally Resize

In [3]:

```
"""
All image sets are read using the cv2.imread method and resized to a predefined
(desired) image width and height. This
ensures all images were equally scaled
"""

# Specify the desired width and height
desired_width = 240
desired_height = 235

# Read first image set
img1 = cv2.imread(img_path1a)
img1 = cv2.resize(img1, (desired_width, desired_height))
img2 = cv2.imread(img_path1b)
img2 = cv2.resize(img2, (desired_width, desired_height))
img3 = cv2.imread(img_path1c)
img3 = cv2.resize(img3, (desired_width, desired_height))

img4 = cv2.imread(img_path2a)
img4 = cv2.resize(img4, (desired_width, desired_height))
img5 = cv2.imread(img_path2b)
```

```
img5 = cv2.resize(img5, (desired_width, desired_height))
img6 = cv2.imread(img_path2c)
img6 = cv2.resize(img6, (desired_width, desired_height))

# Read second image set
img1r = cv2.imread(img_pathR1)
img1r = cv2.resize(img1r, (desired_width, desired_height))
img2r = cv2.imread(img_pathR2)
img2r = cv2.resize(img2r, (desired_width, desired_height))

# Read third image set
img1b = cv2.imread(img_path1A)
img1b = cv2.resize(img1b, (desired_width, desired_height))
img2b = cv2.imread(img_path1B)
img2b = cv2.resize(img2b, (desired_width, desired_height))

img3b = cv2.imread(img_path1C)
img3b = cv2.resize(img3b, (desired_width, desired_height))
img4b = cv2.imread(img_path2A)
img4b = cv2.resize(img4b, (desired_width, desired_height))

img5b = cv2.imread(img_path2B)
img5b = cv2.resize(img5b, (desired_width, desired_height))
img6b = cv2.imread(img_path2C)
img6b = cv2.resize(img6b, (desired_width, desired_height))
```

Convert to HSV (Hue Saturation Value)

In [4]:

```
"""
The images are converted to Hue, Saturation and Value (HSV) which affords colour
representation of images in three
dimensions of rows, columns and color channels.

"""

# Convert image to HSV color
hsv1 = cv2.cvtColor(img1, cv2.COLOR_BGR2HSV)
hsv2 = cv2.cvtColor(img2, cv2.COLOR_BGR2HSV)
hsv3 = cv2.cvtColor(img3, cv2.COLOR_BGR2HSV)
hsv4 = cv2.cvtColor(img4, cv2.COLOR_BGR2HSV)
hsv5 = cv2.cvtColor(img5, cv2.COLOR_BGR2HSV)
hsv6 = cv2.cvtColor(img6, cv2.COLOR_BGR2HSV)

hsv1r = cv2.cvtColor(img1r, cv2.COLOR_BGR2HSV)
hsv2r = cv2.cvtColor(img2r, cv2.COLOR_BGR2HSV)

hsv1b = cv2.cvtColor(img1b, cv2.COLOR_BGR2HSV)
hsv2b = cv2.cvtColor(img2b, cv2.COLOR_BGR2HSV)
hsv3b = cv2.cvtColor(img3b, cv2.COLOR_BGR2HSV)
hsv4b = cv2.cvtColor(img4b, cv2.COLOR_BGR2HSV)
hsv5b = cv2.cvtColor(img5b, cv2.COLOR_BGR2HSV)
hsv6b = cv2.cvtColor(img6b, cv2.COLOR_BGR2HSV)
```

Check HSV Components

Set Bounds for Blue and Green Hue as well as a Threshold

In [5]:

```
"""
The colour values of the HSV-transformed images are adjusted for fine-grained control by defining an upper and lower colour
range boundaries for green and blue hues respectively. A threshold cutoff value of 37 was applied using the
threshold-binary-inverse method.
"""

# Define the lower and upper bounds for blue color in HSV
lower_blue = np.array([71, 71, 71])  # Adjust these values as needed
upper_blue = np.array([255, 255, 255])

lower_green = np.array([71, 71, 71])  # Adjust these values as needed
upper_green = np.array([255, 255, 255])

# Threshold the images based on the blue and green color ranges
threshold_value = 37
max_value = 255

_, background1 = cv2.threshold(cv2.inRange(hsv1, lower_blue, upper_blue), threshold_value, max_value, cv2.THRESH_BINARY_INV)
_, background2 = cv2.threshold(cv2.inRange(hsv2, lower_blue, upper_blue), threshold_value, max_value, cv2.THRESH_BINARY_INV)
_, background3 = cv2.threshold(cv2.inRange(hsv3, lower_green, upper_green), threshold_value, max_value, cv2.THRESH_BINARY_INV)
_, background4 = cv2.threshold(cv2.inRange(hsv4, lower_blue, upper_blue), threshold_value, max_value, cv2.THRESH_BINARY_INV)
_, background5 = cv2.threshold(cv2.inRange(hsv5, lower_blue, upper_blue), threshold_value, max_value, cv2.THRESH_BINARY_INV)
_, background6 = cv2.threshold(cv2.inRange(hsv6, lower_green, upper_green), threshold_value, max_value, cv2.THRESH_BINARY_INV)

_, background1r = cv2.threshold(cv2.inRange(hsv1r, lower_blue, upper_blue), threshold_value, max_value, cv2.THRESH_BINARY_INV)
_, background2r = cv2.threshold(cv2.inRange(hsv2r, lower_blue, upper_blue), threshold_value, max_value, cv2.THRESH_BINARY_INV)


_, background1b = cv2.threshold(cv2.inRange(hsv1b, lower_blue, upper_blue), threshold_value, max_value, cv2.THRESH_BINARY_INV)
_, background2b = cv2.threshold(cv2.inRange(hsv2b, lower_blue, upper_blue), threshold_value, max_value, cv2.THRESH_BINARY_INV)
_, background3b = cv2.threshold(cv2.inRange(hsv3b, lower_green, upper_green), threshold_value, max_value, cv2.THRESH_BINARY_INV)
_, background4b = cv2.threshold(cv2.inRange(hsv4b, lower_blue, upper_blue), threshold_value, max_value, cv2.THRESH_BINARY_INV)
_, background5b = cv2.threshold(cv2.inRange(hsv5b, lower_blue, upper_blue), threshold_value, max_value, cv2.THRESH_BINARY_INV)
_, background6b = cv2.threshold(cv2.inRange(hsv6b, lower_green, upper_green), threshold_value, max_value, cv2.THRESH_BINARY_INV)
```

Calculate the Respective Contours

In [6]:

```
"""
The Chain-Approximate-Simple method is applied to compress horizontal, vertical,
and diagonal segments. The result is a
NumPy array of x- and y-coordinates of contour points
"""

# Find Contours
contours1, _ = cv2.findContours(background1, cv2.RETR_EXTERNAL,
cv2.CHAIN_APPROX_SIMPLE)
contours2, _ = cv2.findContours(background2, cv2.RETR_EXTERNAL,
cv2.CHAIN_APPROX_SIMPLE)
contours3, _ = cv2.findContours(background3, cv2.RETR_EXTERNAL,
cv2.CHAIN_APPROX_SIMPLE)
contours4, _ = cv2.findContours(background4, cv2.RETR_EXTERNAL,
cv2.CHAIN_APPROX_SIMPLE)
contours5, _ = cv2.findContours(background5, cv2.RETR_EXTERNAL,
cv2.CHAIN_APPROX_SIMPLE)
contours6, _ = cv2.findContours(background6, cv2.RETR_EXTERNAL,
cv2.CHAIN_APPROX_SIMPLE)

contours1r, _ = cv2.findContours(background1r, cv2.RETR_EXTERNAL,
cv2.CHAIN_APPROX_SIMPLE)
contours2r, _ = cv2.findContours(background2r, cv2.RETR_EXTERNAL,
cv2.CHAIN_APPROX_SIMPLE)

contours1b, _ = cv2.findContours(background1b, cv2.RETR_EXTERNAL,
cv2.CHAIN_APPROX_SIMPLE)
contours2b, _ = cv2.findContours(background2b, cv2.RETR_EXTERNAL,
cv2.CHAIN_APPROX_SIMPLE)
contours3b, _ = cv2.findContours(background3b, cv2.RETR_EXTERNAL,
cv2.CHAIN_APPROX_SIMPLE)
contours4b, _ = cv2.findContours(background4b, cv2.RETR_EXTERNAL,
cv2.CHAIN_APPROX_SIMPLE)
contours5b, _ = cv2.findContours(background5b, cv2.RETR_EXTERNAL,
cv2.CHAIN_APPROX_SIMPLE)
contours6b, _ = cv2.findContours(background6b, cv2.RETR_EXTERNAL,
cv2.CHAIN_APPROX_SIMPLE)
```

Find Area of Contours for Each Image

In [7]:

```
"""
The combined area of all estimated contours is calculated in pixel squared unit
- a measure of the bioluminescence intensity.
"""

# Quantification
# Calculate total area of transduced contours
# Bioluminescent image example 1
total_area1 = 0
total_area2 = 0
total_area3 = 0
```

```python
total_area4 = 0
total_area5 = 0
total_area6 = 0

# Bioluminescent image example 2
total_area1r = 0
total_area2r = 0

# Luciferase images
total_area1b = 0
total_area2b = 0
total_area3b = 0
total_area4b = 0
total_area5b = 0
total_area6b = 0

# Bioluminescent image example 1
for cnt1 in contours1:
    area1 = cv2.contourArea(cnt1)
    total_area1 += area1

for cnt2 in contours2:
    area2 = cv2.contourArea(cnt2)
    total_area2 += area2

for cnt3 in contours3:
    area3 = cv2.contourArea(cnt3)
    total_area3 += area3

for cnt4 in contours4:
    area4 = cv2.contourArea(cnt4)
    total_area4 += area4

for cnt5 in contours5:
    area5 = cv2.contourArea(cnt5)
    total_area5 += area5

for cnt6 in contours6:
    area6 = cv2.contourArea(cnt6)
    total_area6 += area6

# Bioluminescent image example 2
for cnt1r in contours1r:
    area1r = cv2.contourArea(cnt1r)
    total_area1r += area1r

for cnt2r in contours2r:
    area2r = cv2.contourArea(cnt2r)
    total_area2r += area2r

# Luciferase images
for cnt1b in contours1b:
    area1b = cv2.contourArea(cnt1b)
    total_area1b += area1b

for cnt2b in contours2b:
    area2b = cv2.contourArea(cnt2b)
    total_area2b += area2b
```

```
for cnt3b in contours3b:
    area3b = cv2.contourArea(cnt3b)
    total_area3b += area3b

for cnt4b in contours4b:
    area4b = cv2.contourArea(cnt4b)
    total_area4b += area4b

for cnt5b in contours5b:
    area5b = cv2.contourArea(cnt5b)
    total_area5b += area5b

for cnt6b in contours6b:
    area6b = cv2.contourArea(cnt6b)
    total_area6b += area6b

# Output: total_area represents the total bioluminescent area
print('The area 1st image: {} pixels squared'.format(total_area1))
print('The area 2nd image: {} pixels squared'.format(total_area2))
print('The area 3rd image: {} pixels squared'.format(total_area3))
print('The area 4th image: {} pixels squared'.format(total_area4))
print('The area 5th image: {} pixels squared'.format(total_area5))
print('The area 6th image: {} pixels squared'.format(total_area6))

print('The area of 1st image: {} pixels squared'.format(total_area1r))
print('The area of 2nd image: {} pixels squared'.format(total_area2r))


print('The area of 1st image: {} pixels squared'.format(total_area1b))
print('The area of 2nd image: {} pixels squared'.format(total_area2b))
print('The area of 3rd image: {} pixels squared'.format(total_area3b))
print('The area of 4th image: {} pixels squared'.format(total_area4b))
print('The area of 5th image: {} pixels squared'.format(total_area5b))
print('The area of 6th image: {} pixels squared'.format(total_area6b))
```

```
The area 1st image: 1.0 pixels squared
The area 2nd image: 0.0 pixels squared
The area 3rd image: 230.5 pixels squared
The area 4th image: 833.5 pixels squared
The area 5th image: 0.0 pixels squared
The area 6th image: 12.5 pixels squared
The area of 1st image: 297.5 pixels squared
The area of 2nd image: 2.5 pixels squared
The area of 1st image: 0 pixels squared
The area of 2nd image: 212.0 pixels squared
The area of 3rd image: 15182.5 pixels squared
The area of 4th image: 0 pixels squared
The area of 5th image: 569.5 pixels squared
The area of 6th image: 16889.0 pixels squared
```

PLOT THE GRAPH

Bioluminescent Image Example 1

In [8]:

```
"""
The computed pixel bioluminescent intensities of the various image sets are
plotted graphically.
"""

# Create the DataFrame
data = {
    "Image": ['1st_image','2nd_image','3rd_image','4th_image',
'5th_image','6th_image'],
    "Contour": [total_area1, total_area2, total_area3, total_area4, total_area5,
total_area6],  # Fill the 'positive' column with zeros
    "Category": ['Control','Control','Transduced','Transduced',
'Negative_control','Negative_control'],
    }


df = pd.DataFrame(data)

# Sample data
categories = ['Control_control','Control_Positive_1', 'Control_Positive_2']
values_group1 = [total_area1,total_area6, total_area5]
values_group2 = [total_area2,total_area3, total_area4]

# Define the width of the bars
bar_width = 0.6

# Set the position of the bars on the x-axis
x = np.arange(len(categories))

# Create the grouped bar plot
plt.bar(x - bar_width/2, values_group1, width=bar_width, color='grey',
label='Control')#b48663, #532729,#834d1e
plt.bar(x + bar_width/2, values_group2, width=bar_width, color='#b48663',
label='Positive')

threshold = 37
plt.axhline(y=threshold, color='blue', linestyle='--', linewidth=1.5,
label='Threshold (pixel=37)')

# Add labels, title, and legend
plt.xlabel('Images')
plt.ylabel('Area of Contour (pixel squared)')
plt.suptitle('A', x=0.05, y=0.95)
plt.title('Quantification Plot of Bioluminescence Intensity')
plt.xticks(x, categories)
plt.grid(axis='y', linestyle='--', alpha=0.7)
plt.legend(loc='best', bbox_to_anchor=(0.77, 0., 0.65, 1.02))

plt.show()
```

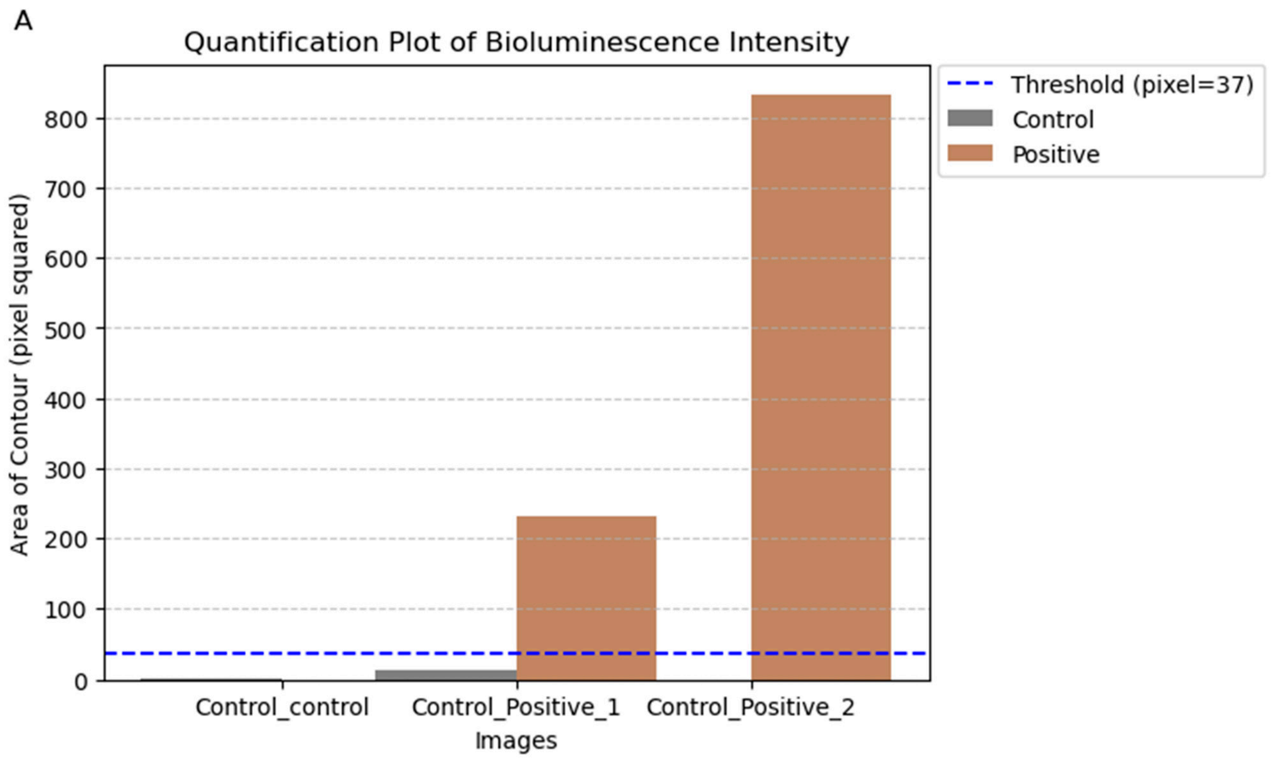

Bioluminescent Image Example 2

In [9]:

```
# Sample data
categories = ['Control_Positive']
values_group1 = [total_area1r]
values_group2 = [total_area2r]

# Define the width of the bars
bar_width = 0.1

# Set the position of the bars on the x-axis
x = np.arange(len(categories))

# Create the grouped bar plot with an offset between pairs
offset = 0.09
plt.bar(x - offset, values_group1, width=bar_width, color='#b48663',
label='Positive')
plt.bar(x + offset, values_group2, width=bar_width, color='grey',
label='Control')#b48663, #532729,#834d1e

# Add a dotted line at y-axis = 37
threshold = 37
plt.axhline(y=threshold, color='blue', linestyle='--', linewidth=1.5,
label='Threshold (pixel=37)')

# Add labels, title, and legend
plt.xlabel('Images')
plt.ylabel('Area of Contour (pixel squared)')
plt.suptitle('B', x=0.05, y=0.95)
plt.title('Quantification Plot of Bioluminescence Intensity')
```

```
plt.xticks(x, categories)
plt.grid(axis='y', linestyle='--', alpha=0.7)
plt.legend(loc='best', bbox_to_anchor=(0.77, 0., 0.65, 1.02))

plt.show()
```

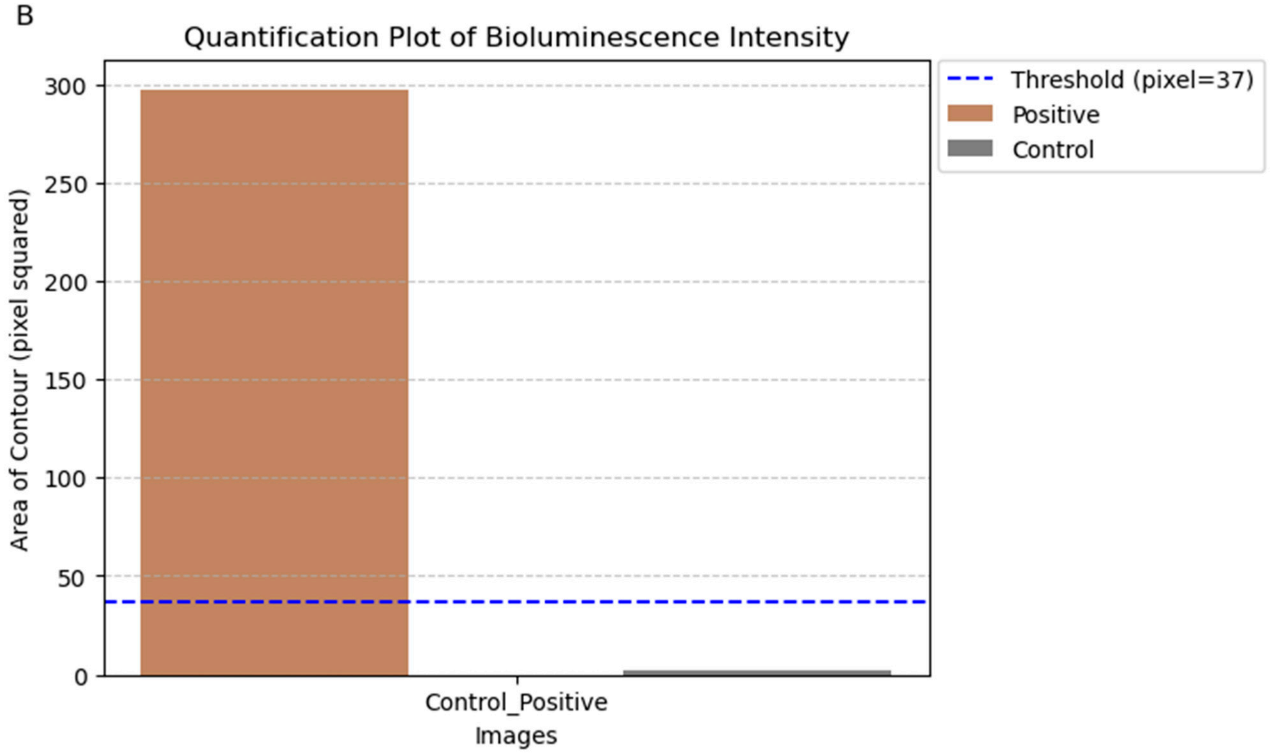

Luciferase Images

In [10]:

```
# Create the DataFrame
datab = {
    "Image": ['1st_image','2nd_image','3rd_image','4th_image',
'5th_image','6th_image'],
    "Contour": [total_area1b, total_area2b, total_area3b, total_area4b,
total_area5b, total_area6b],  # Fill the 'positive' column with zeros
    "Category": ['Control','Transduced_1MOI','Transduced_10MOI','Control',
'Transduced_1MOI','Transduced_10MOI'],
    }

dfb = pd.DataFrame(datab)

# Plot Barplot
plt.figure(figsize=(9, 6))

# Sample data
categories = ['Negative_Control', 'Transduced_1MOI','Transduced_10MOI']
values_group1 = [total_area1b, total_area2b, total_area3b]
values_group2 = [total_area4b, total_area5b, total_area6b]

# Define the width of the bars
bar_width = 0.395
```

```
# Set the position of the bars on the x-axis
x = np.arange(len(categories))

# Create the grouped bar plot with an offset between pairs
offset = 0.2
plt.bar(x - offset, values_group1, width=bar_width, color='#b48663',
label='First example')
plt.bar(x + offset, values_group2, width=bar_width, color='#834d1e',
label='Second example')#b48663, #532729,#834d1e

threshold = 37
plt.axhline(y=threshold, color='blue', linestyle='--', linewidth=1.5,
label='Threshold (pixel=37)')

# Add labels, title, and legend
plt.xlabel('Images')
plt.ylabel('Area of Contour (pixel squared)')
plt.suptitle('C', x=0.05, y=0.95)
plt.title('Quantification of Luciferase Intensity')
plt.xticks(x, categories) # rotation=45, ha='right'
plt.grid(axis='y', linestyle='--', alpha=0.7)
plt.legend(loc='best', bbox_to_anchor=(0.77, 0., 0.526, 1.017))
plt.show()
```

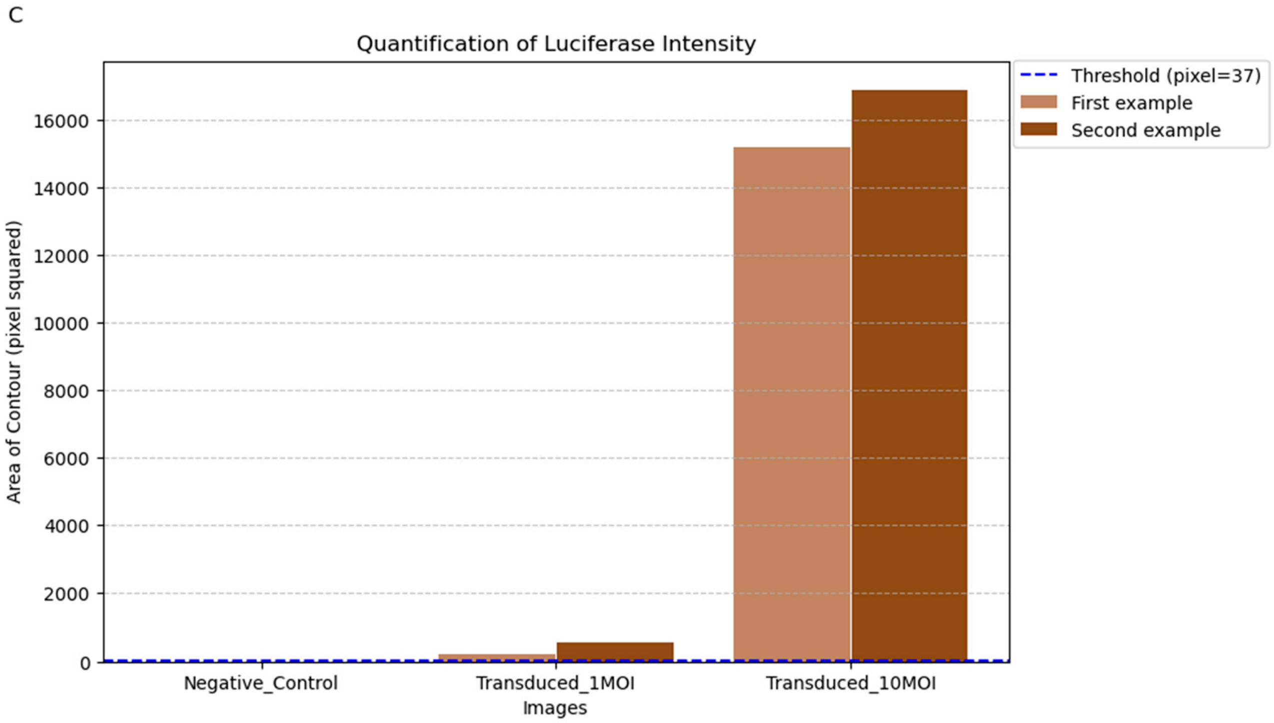

Image Visualization

In [11]:

```
"""
The calculated bioluminescence intensity for each image is validated by first
Gray-transforming images into two-dimensional
NumPy arrays and the previously stated threshold applied.
"""
```

```
# convert to images to grey and scale for first set of images
grey1 = cv2.cvtColor(img1, cv2.COLOR_BGR2GRAY)
grey2 = cv2.cvtColor(img2, cv2.COLOR_BGR2GRAY)

grey3 = cv2.cvtColor(img3, cv2.COLOR_BGR2GRAY)
grey4 = cv2.cvtColor(img4, cv2.COLOR_BGR2GRAY)

grey5 = cv2.cvtColor(img5, cv2.COLOR_BGR2GRAY)
grey6 = cv2.cvtColor(img6, cv2.COLOR_BGR2GRAY)

# convert to images to grey and scale for second set of images
grey1r = cv2.cvtColor(img1r, cv2.COLOR_BGR2GRAY)
grey2r = cv2.cvtColor(img2r, cv2.COLOR_BGR2GRAY)


# convert to images to grey and scale for third set of images
grey1b = cv2.cvtColor(img1b, cv2.COLOR_BGR2GRAY)
grey2b = cv2.cvtColor(img2b, cv2.COLOR_BGR2GRAY)

grey3b = cv2.cvtColor(img3b, cv2.COLOR_BGR2GRAY)
grey4b = cv2.cvtColor(img4b, cv2.COLOR_BGR2GRAY)

grey5b = cv2.cvtColor(img5b, cv2.COLOR_BGR2GRAY)
grey6b = cv2.cvtColor(img6b, cv2.COLOR_BGR2GRAY)

# Check that all images have been equally scaled
print('shape of 1st example transduced grey image is {}'.format(grey1.shape))
print('shape of 2nd example control grey image is {}'.format(grey4.shape))

print('shape of 1st example transduced grey image is {}'.format(grey1r.shape))
print('shape of 2nd example control grey image is {}'.format(grey2r.shape))

print('shape of 1st example transduced grey image is {}'.format(grey1b.shape))
print('shape of 2nd eexample control grey image is {}'.format(grey4b.shape))
```

```
shape of 1st example transduced grey image is (235, 240)
shape of 2nd example control grey image is (235, 240)
shape of 1st example transduced grey image is (235, 240)
shape of 2nd example control grey image is (235, 240)
shape of 1st example transduced grey image is (235, 240)
shape of 2nd eexample control grey image is (235, 240)
```

In [12]:

```
# Place a background subtraction method for first set of images
_, background1 = cv2.threshold(grey1, 37, 255, cv2.THRESH_BINARY_INV)
_, background2 = cv2.threshold(grey2, 37, 255, cv2.THRESH_BINARY_INV)

_, background3 = cv2.threshold(grey3, 37, 255, cv2.THRESH_BINARY_INV)
_, background4 = cv2.threshold(grey4, 37, 255, cv2.THRESH_BINARY_INV)

_, background5 = cv2.threshold(grey5, 37, 255, cv2.THRESH_BINARY_INV)
_, background6 = cv2.threshold(grey6, 37, 255, cv2.THRESH_BINARY_INV)

# Place a background subtraction method for second set of images
_, background1r = cv2.threshold(grey1r, 37, 255, cv2.THRESH_BINARY_INV)
_, background2r = cv2.threshold(grey2r, 37, 255, cv2.THRESH_BINARY_INV)
```

First Image Set

In [13]:

```
"""
The original, grey-transformed, and without grey filter images are compared to
ensure accuracy of bioluminescent intensity.
"""

# view first set of images
fig, axes = plt.subplots(nrows=2, ncols=3, figsize=(8,5))
# First row
axes[0,0].imshow(cv2.cvtColor(img1, cv2.COLOR_BGR2RGB))
axes[0,0].text(-0.05, 0.9, 'A', fontsize=12, transform=axes[0,0].transAxes,
va='center', ha='right')
axes[0,1].imshow(cv2.cvtColor(img2, cv2.COLOR_BGR2RGB))
axes[0,2].imshow(cv2.cvtColor(img3, cv2.COLOR_BGR2RGB))

# Second row
axes[1,0].imshow(cv2.cvtColor(img4, cv2.COLOR_BGR2RGB))
axes[1,1].imshow(cv2.cvtColor(img5, cv2.COLOR_BGR2RGB))
axes[1,2].imshow(cv2.cvtColor(img6, cv2.COLOR_BGR2RGB))

for ax in axes.flat:
    ax.axis('off')

# Add a broad title above the subplots
fig.suptitle('Positive Bioluminescent and Control Images', fontsize=13)

plt.show()
```

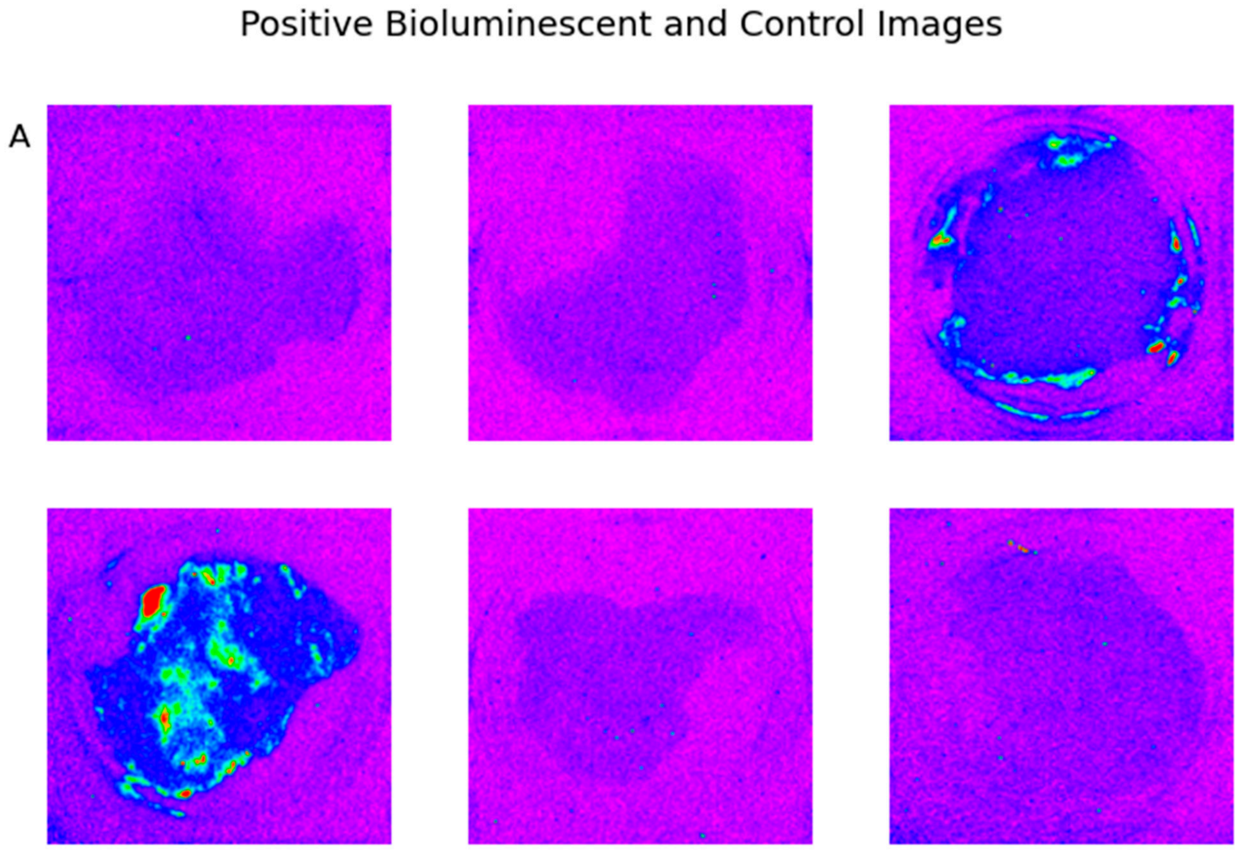

In [14]:

```
# view first set of images WITH gray scale filter
fig, axes = plt.subplots(nrows=2, ncols=3, figsize=(8,5))
# first row
```

```
axes[0,0].imshow(background1, cmap='gray')
axes[0,0].text(-0.05, 0.9, 'A', fontsize=12, transform=axes[0,0].transAxes,
va='center', ha='right')

axes[0,1].imshow(background2, cmap='gray')
axes[0,2].imshow(background3, cmap='gray')

# second row
axes[1,0].imshow(background4, cmap='gray')
axes[1,1].imshow(background5, cmap='gray')
axes[1,2].imshow(background6, cmap='gray')

for ax in axes.flat:
    ax.axis('off')

# Add a broad title above the subplots
fig.suptitle('Gray-Scaled Bioluminescent Images', fontsize=13)

plt.show()

plt.tight_layout()
```

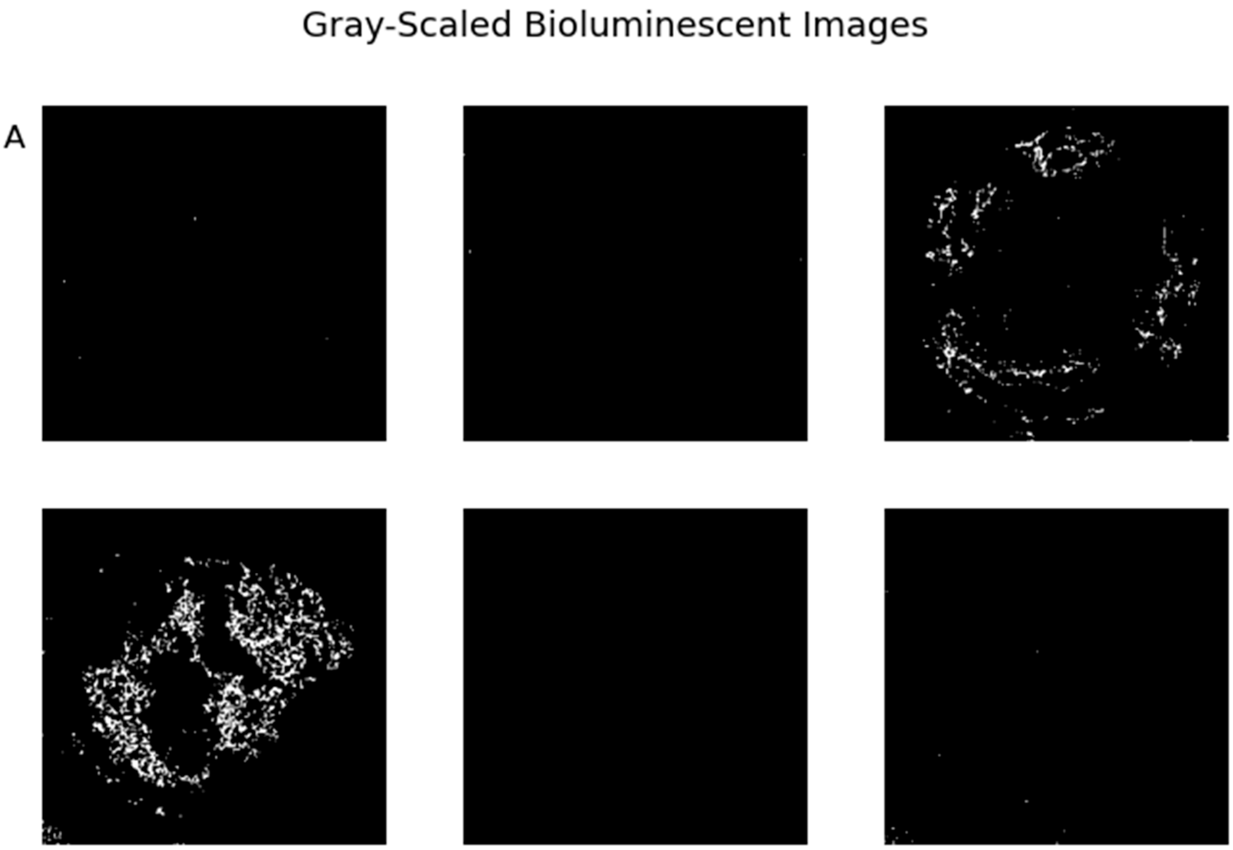


```
<Figure size 640x480 with 0 Axes>
```

In [15]:

```
# view first set of images WITHOUT gray scale filter
fig, axes = plt.subplots(nrows=2, ncols=3, figsize=(8,5))
# first row
axes[0,0].imshow(background1)
axes[0,0].text(-0.05, 0.9, 'A', fontsize=12, transform=axes[0,0].transAxes,
va='center', ha='right')
axes[0,1].imshow(background2)
axes[0,2].imshow(background3)
```

```
# second row
axes[1,0].imshow(background4)
axes[1,1].imshow(background5)
axes[1,2].imshow(background6)

for ax in axes.flat:
    ax.axis('off')

# Add a broad title above the subplots
fig.suptitle('Identified Threshold-Specified Region of Interest', fontsize=13)

# Save the image with best quality
#plt.savefig('positive_control_gray_thresh.png', dpi=300, bbox_inches='tight')

plt.show()

plt.tight_layout()
```

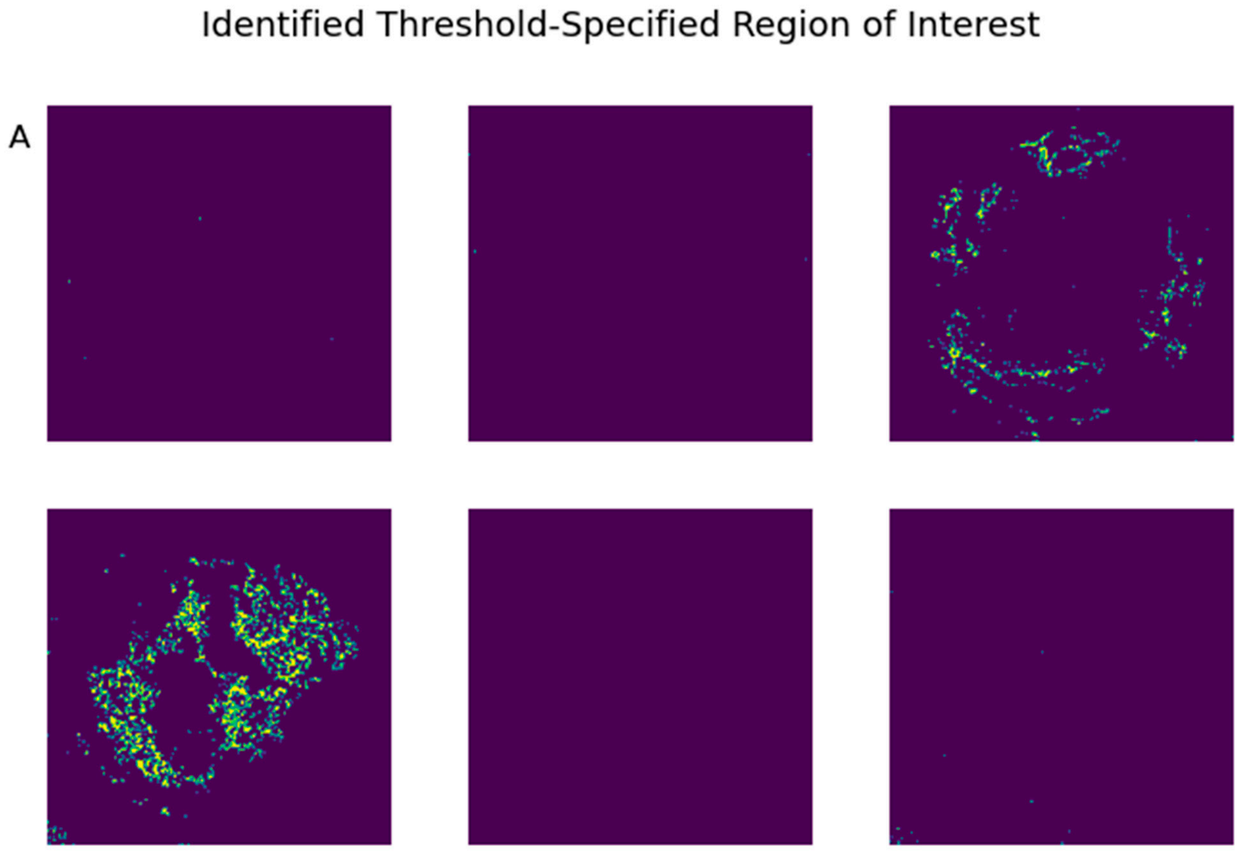


```
<Figure size 640x480 with 0 Axes>
```

Second Image Set

In [16]:

```
# view second set of images
fig, axes = plt.subplots(ncols=2, figsize=(7,3))
axes[0].imshow(cv2.cvtColor(img1r, cv2.COLOR_BGR2RGB))
axes[0].text(-0.05, 0.9, 'B', fontsize=12, transform=axes[0].transAxes,
va='center', ha='right')
```

```
axes[1].imshow(cv2.cvtColor(img2r, cv2.COLOR_BGR2RGB))

for ax in axes.flat:
    ax.axis('off')

plt.show()
```

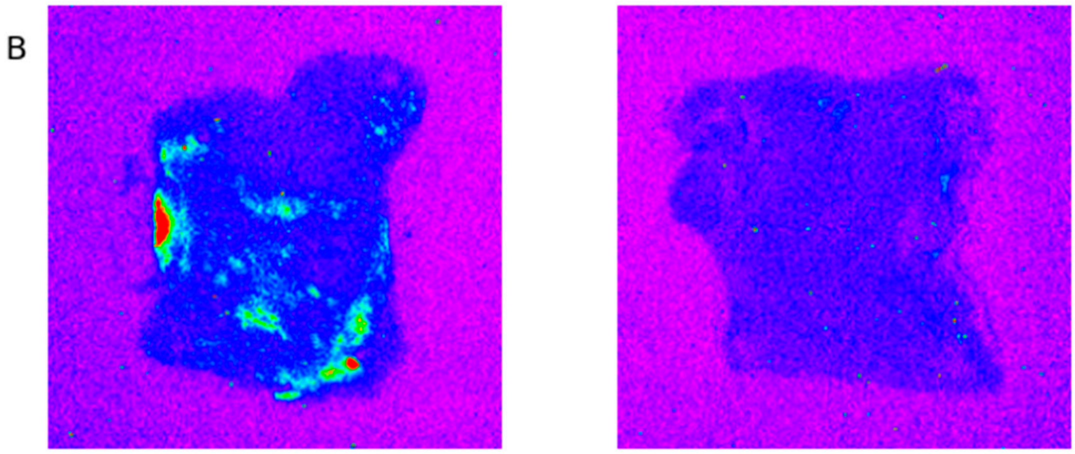

In [17]:

```
# view first set of images WITH gray scale filter
fig, axes = plt.subplots(ncols=2, figsize=(7,3))
# first row
axes[0].imshow(background1r, cmap='gray')
axes[0].text(-0.05, 0.9, 'B', fontsize=12, transform=axes[0].transAxes,
va='center', ha='right')
axes[1].imshow(background2r, cmap='gray')

for ax in axes.flat:
    ax.axis('off')


plt.show()
```

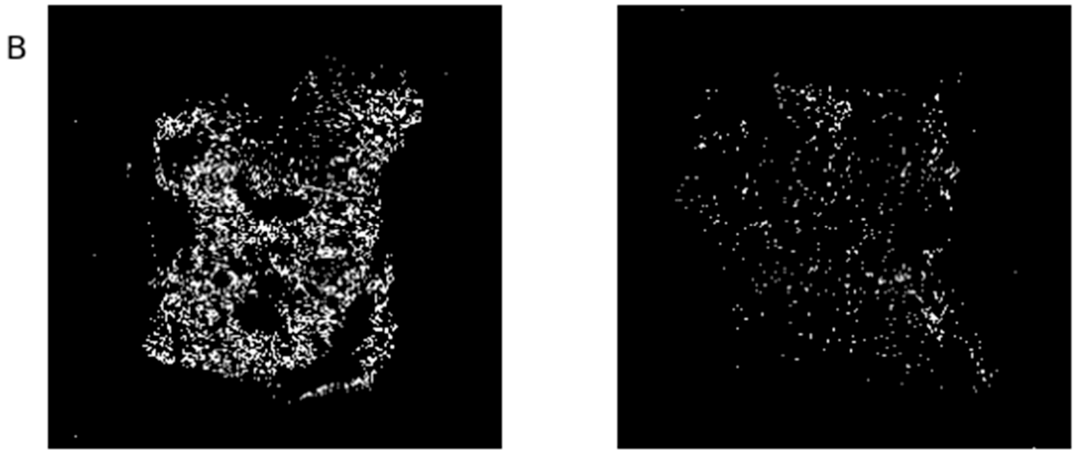

In [18]:

```
# view first set of images WITH gray scale filter
fig, axes = plt.subplots(ncols=2, figsize=(7,3))
# first row
axes[0].imshow(background1r)
axes[0].text(-0.05, 0.9, 'B', fontsize=12, transform=axes[0].transAxes,
va='center', ha='right')
```

```
axes[1].imshow(background2r)

for ax in axes.flat:
    ax.axis('off')

plt.show()
```

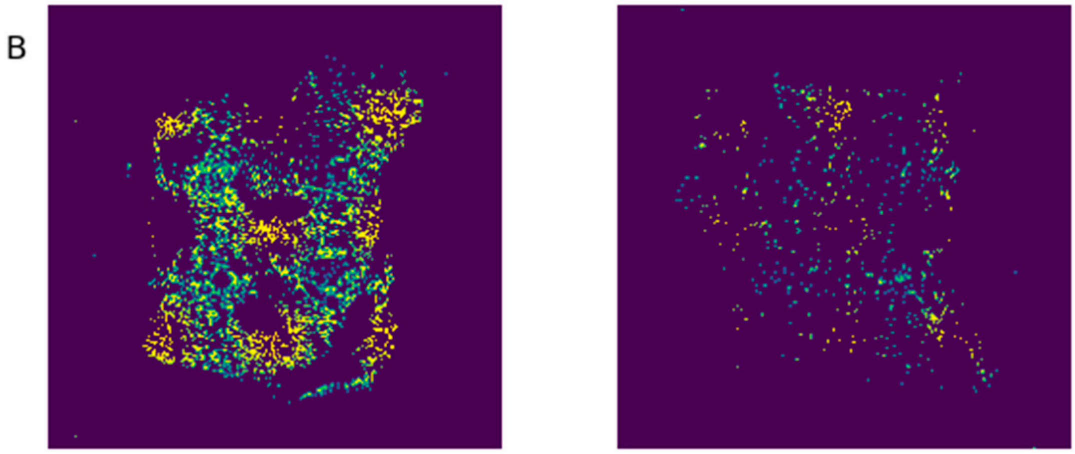

Luciferase Intensity and Quantification

In [19]:

```
# view third set of images
fig, axes = plt.subplots(nrows=2, ncols=3, figsize=(8,5))
# First row
axes[0,0].imshow(cv2.cvtColor(img1b, cv2.COLOR_BGR2RGB))
axes[0,0].text(-0.05, 0.9, 'C', fontsize=12, transform=axes[0,0].transAxes,
va='center', ha='right')

axes[0,1].imshow(cv2.cvtColor(img2b, cv2.COLOR_BGR2RGB))
axes[0,2].imshow(cv2.cvtColor(img3b, cv2.COLOR_BGR2RGB))

# Second row
axes[1,0].imshow(cv2.cvtColor(img4b, cv2.COLOR_BGR2RGB))
axes[1,1].imshow(cv2.cvtColor(img5b, cv2.COLOR_BGR2RGB))
axes[1,2].imshow(cv2.cvtColor(img6b, cv2.COLOR_BGR2RGB))

for ax in axes.flat:
    ax.axis('off')

plt.show()
```

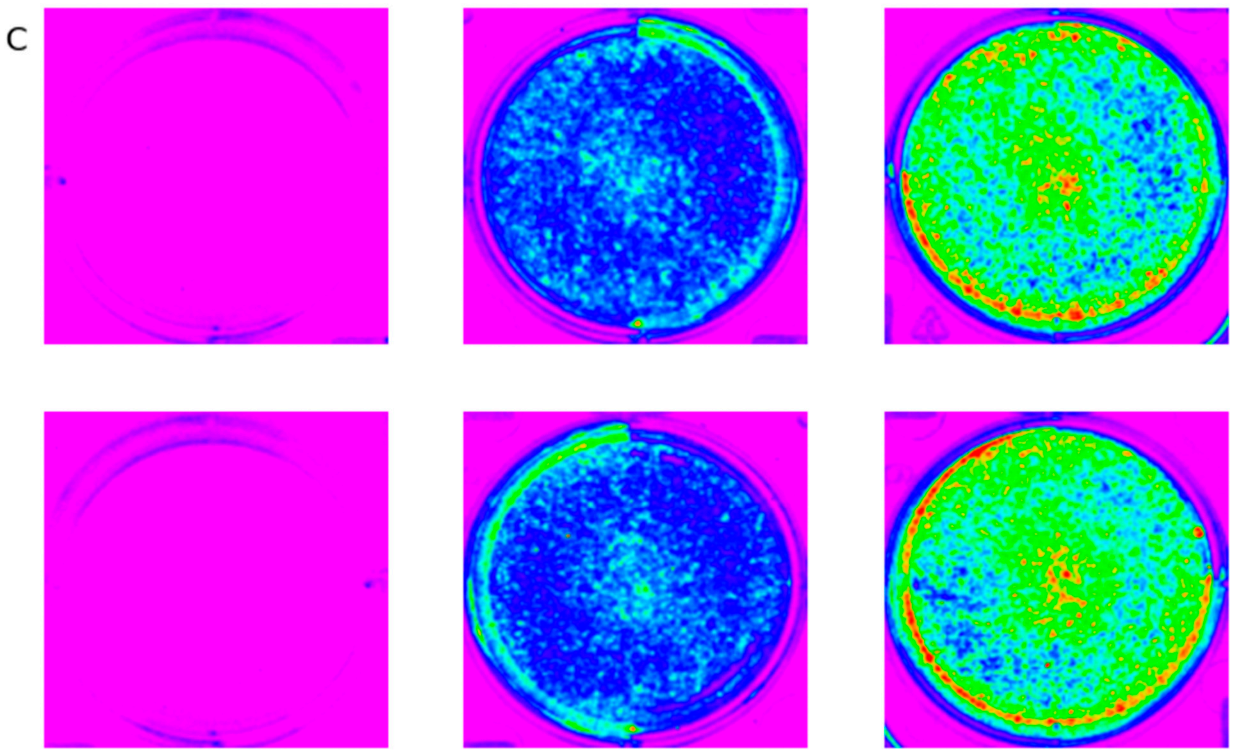

In [20]:

```
# view first set of images WITH gray scale filter
fig, axes = plt.subplots(nrows=2, ncols=3, figsize=(8,5))
# first row
axes[0,0].imshow(background1b, cmap='gray')
axes[0,0].text(-0.05, 0.9, 'C', fontsize=12, transform=axes[0,0].transAxes,
va='center', ha='right')
axes[0,1].imshow(background2b, cmap='gray')
axes[0,2].imshow(background3b, cmap='gray')

# second row
axes[1,0].imshow(background4b, cmap='gray')
axes[1,1].imshow(background5b, cmap='gray')
axes[1,2].imshow(background6b, cmap='gray')

for ax in axes.flat:
    ax.axis('off')


plt.show()
```

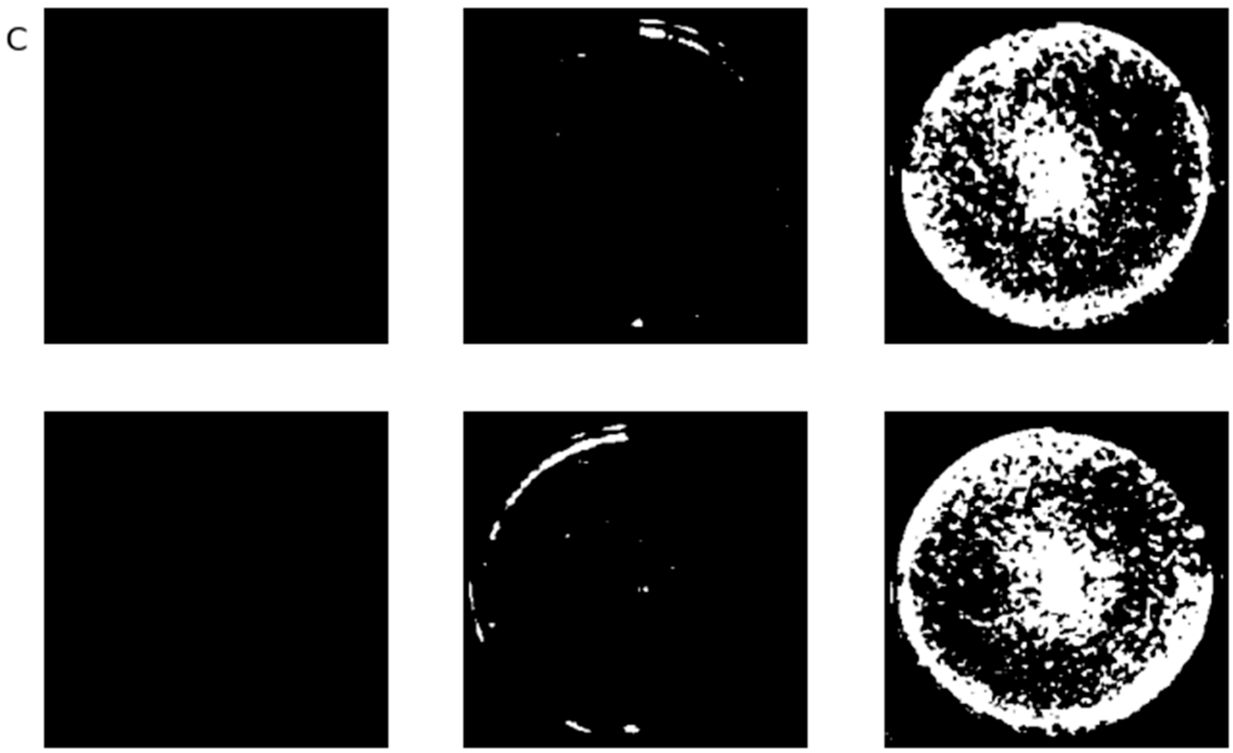

In [21]:

```
# view first set of images WITHOUT gray scale filter
fig, axes = plt.subplots(nrows=2, ncols=3, figsize=(8,5))
# first row
axes[0,0].imshow(background1b)
axes[0,0].text(-0.05, 0.9, 'C', fontsize=12, transform=axes[0,0].transAxes,
va='center', ha='right')
axes[0,1].imshow(background2b)
axes[0,2].imshow(background3b)

# second row
axes[1,0].imshow(background4b)
axes[1,1].imshow(background5b)
axes[1,2].imshow(background6b)

for ax in axes.flat:
    ax.axis('off')

plt.show()
```

C

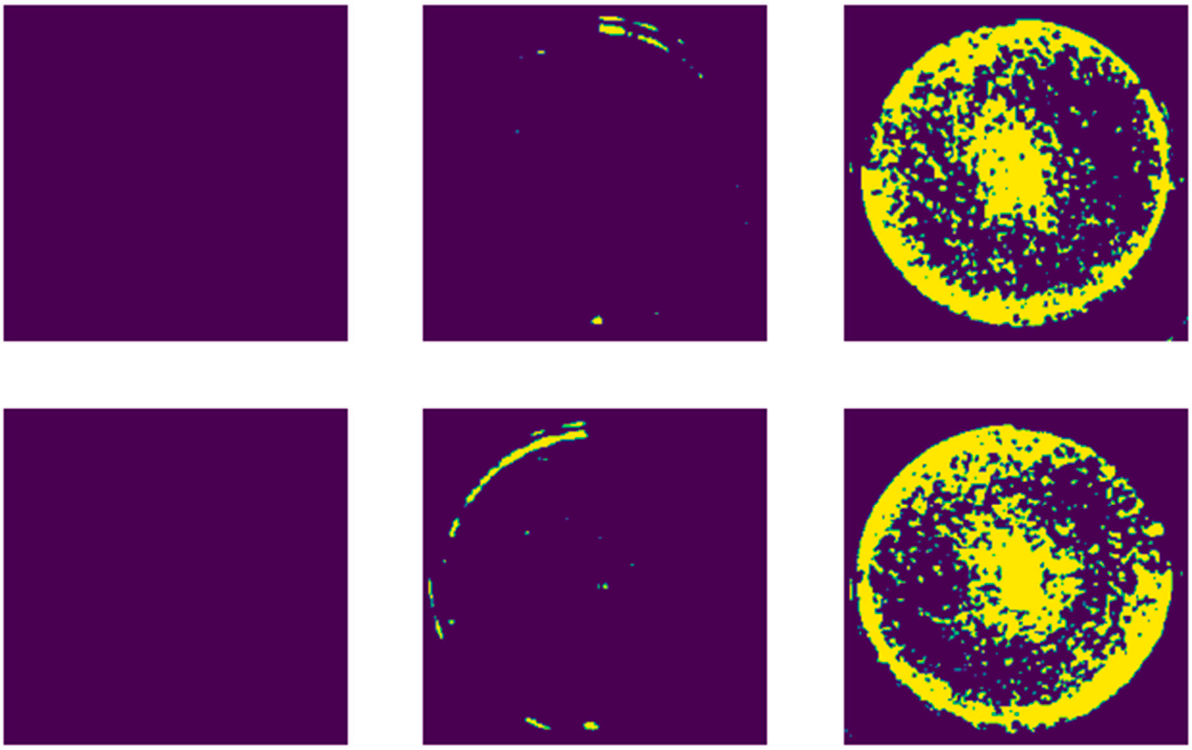

Supplement: Supplementary file 1 [file vaccines-12-00851-s001.zip › vaccines-3113636-supplementary.pdf]
